# Supplementary material for: Identification of photoperiod-induced specific miRNAs in the adrenal glands of Sunite sheep (Ovis aries)
Source: Front Vet Sci. 2022 Jul 22;9:888207. doi: 10.3389/fvets.2022.888207 (PMC9354845; doi:10.3389/fvets.2022.888207)
Supplement: Supplementary file 1 [file Data_Sheet_1.ZIP › Supplementary file/miRDeep2 file.zip!/miRDeep2 file/Novel_Structure/SPLP42A2/Novel_189.pdf]

Provisional ID	:	NC_019475.2_32899
Score total	:	2.5
Score for star read(s)	:	-1.3
Score for read counts	:	0
Score for mfe	:	2.2
Score for randfold	:	1.6
Score for cons. seed	:	
Total read count	:	148
Mature read count	:	138
Loop read count	:	0
Star read count	:	10

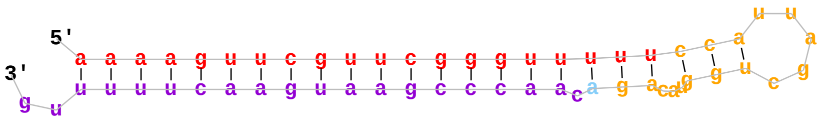

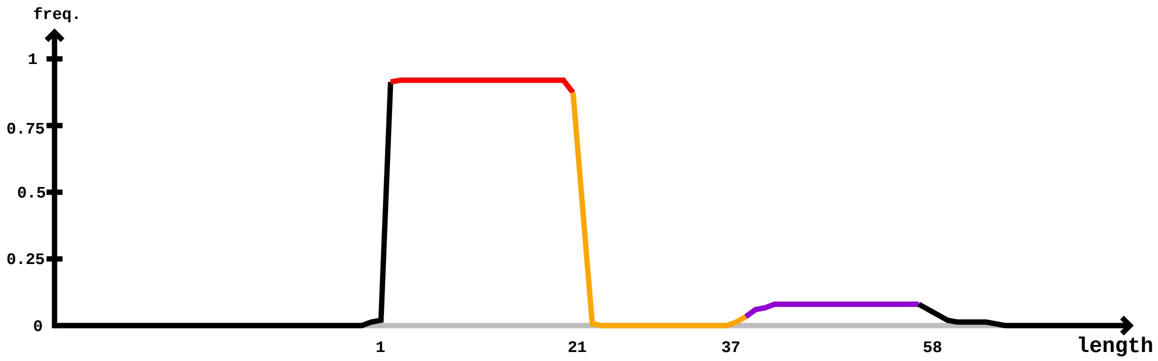


```
                                 Mature                                 Star
5'- ccuggggaggaagggauguacuggguuggcccaaaaguucguucggguuuuuccauuagcugguacagacaacccgaaugaacuuuuuggccaauucaauauuuccagc   -3'   obs
    ccuggggaggaagggauguacuggguuggcccaaaaguucguucggguuuuuccauuagcugguacagacaacccgaaugaacuuuuuggccaauucaauauuuccagc         exp
    (((.....)))...(((.(((.(((((((((.(((((((((((((((((((((((.....)))...))).))))))))))))))))).)))))))))).))).)))...   reads   mm   sample
    ..............................cAaaaaguucguucggguuuuu.........................................................   2       1    seq
    ...............................caaaaguucguucggguuuuuc........................................................   1       0    seq
    ................................aaaaguucguucggguuuu..........................................................   7       0    seq
    ................................aaaaguucguucggguuuuu.........................................................   126     0    seq
    ................................aaaaguucguucggguuuuucc.......................................................   1       0    seq
    .................................aaaguucguucggguuuuuc........................................................   1       0    seq
    ....................................................................acaacccAaaugaacuuuuu.....................   1       1    seq
    ....................................................................acaacccgaaugaacuuuuu.....................   1       0    seq
    .....................................................................caacccgaaugaacuuuuug....................   3       0    seq
    ......................................................................aacccgaaugaacuuuuu.....................   1       0    seq
    ......................................................................aacccgaaugaacuuuuug....................   2       0    seq
    ......................................................................aacccgaaugaacuuuuuC....................   1       1    seq
    .......................................................................acccgaaugaacuuuuuggc..................   1       0    seq
    ........................................................................ccUgaaugaacuuuuuggccaau..............   1       1    seq
    ........................................................................ccUgaaugaacuuuuuggccaauu.............   1       1    seq
```
